# Epigallocatechin-3-gallate suppresses 1-methyl-4-phenyl-pyridine-induced oxidative stress in PC12 cells via the SIRT1/PGC-1α signaling pathway

**DOI:** 10.1186/1472-6882-12-82

**Published:** 2012-06-28

**Authors:** Qinyong Ye, Linfeng Ye, Xianjie Xu, Bixia Huang, Xiaodong Zhang, Yuangui Zhu, Xiaochun Chen

**Affiliations:** 1 Department of Neurology, Fujian Institute of Geriatrics, The Affiliated Union Hospital of Fujian Medical University, 29 Xinquan Road, Fuzhou, Fujian, 350001, China

**Keywords:** Parkinson’s disease, (−)-epigallocatechin-3-gallate, PC12 cells, PGC-1α, SIRT1

## Abstract

**Background:**

Parkinson’s disease is a high incidence neurodegenerative disease in elderly people, and oxidative stress plays an important role in the pathogenesis. Oxygen metabolism in the brain is high, which lacks an antioxidative protection mechanism. Recently, it has been found that polyphenols play an important role in antioxidation. (−)-epigallocatechin-3-gallate (EGCG) is an important component of tea polyphenols and its biological effects, such as strong antioxidation, scavenging of free radicals and anti-apoptosis, can pass through the blood brain barrier. The SIRT1/PGC-1α signaling pathway has not been reported in PC12 cells. Therefore, research of the protective mechanism of EGCG in PC12 cells damaged by -methyl-4-phenyl-pyridine (MMP^+^) may provide a new insight into protect against and treatment of Parkinson’s disease.

**Methods:**

MPP^+^-treated highly differentiated PC12 cells were used as the in vitro cell model. An MTT assay was used to investigate cell viability after EGCG treatment, a dichlorofluorescin diacetate assay was used to measure reactive oxygen species (ROS) production, western blot analysis was used to observe PGC-1α and SIRT1 protein expression, and real-time PCR to observe PGC-1α, SOD1 and GPX1 mRNA expression.

**Results:**

PC12 cell viability was significantly reduced after MPP^+^ treatment by 11.46% compared with that of the control (P < 0.05). However, cell viability was unchanged by 10 μmol/L EGCG treatment. In co-treatments with EGCG and MPP^+^, cell viability was significantly increased by 12.92% (P < 0.05) and MPP^+^-induced ROS production was markedly decreased. PGC-1α mRNA expression was obviously upregulated by 21.51% (P < 0.05), and SOD1 and GPX1 mRNA expression was slightly increased by 12.94% and 15.63% (P > 0.05), respectively, by treatment with EGCG and then MPP^+^ for 12 h. The mRNA expression of PGC-1α, SOD1 and GPX1 was increased by 25.17%, 40% and 146% (all P < 0.05), respectively, by treatment with EGCG and then MPP^+^ for 24 h. Such effects were not observed with MPP^+^ treatment alone.

**Conclusion:**

The SIRT1/PGC-1α pathway is one of the mechanisms of EGCG suppression of MPP^+^-induced injury of PC12 cells.

## Background

Polyphenols are present in green tea extract, and many studies have shown that polyphenols have strong anti-oxidant and -apoptosis activities [1]. Most of the benefits of green tea are due to the most prominent biological activity of the green tea catechin (−)-epigallocatechin-3-gallate (EGCG) [2]. Oxidative stress and cell apoptosis play an important role in the pathogenesis of Parkinson’s disease [3]. Therefore, we considered whether EGCG has a protective effect against Parkinson’s disease. The role of the silent mating type information regulation 2 homolog (SIRT1)/peroxisomal proliferator-activated receptor- coactivator 1 (PGC-1α) signaling pathway in oxidative stress induced by 1-methyl-4-phenyl-pyridine (MPP^+^) in PC12 cells and whether EGCG is antagonized in PC12 cells with MPP^+^-induced oxidative stress via the SIRT1/PGC-1α cell signaling pathway have not been reported. In this study, PC12 cells were used as a model of dopaminergic neurons with MPP^+^-induced cell injury, and the protective effect and mechanism of EGCG suppression of MPP^+^-induced cell injury were analyzed to provide a biological basis for prevention and treatment of Parkinson's disease.

## Methods

### Reagents

Cell culture reagents were purchased from Hyclone (Logan, UT, USA). Acrylamide and western blot reagents were purchased from Bio-Rad (Hercules,California, USA). MPP^+^ and EGCG were purchased from Sigma-Aldrich (St. Louis, MO, USA). Anti-PGC-1α and -SIRT1 antibodies were purchased from Santa Cruz Biotechnology Inc. (Santa Cruz, CA, USA). Anti-actin antibody, goat anti-rabbit and goat anti-mouse IgG were purchased from Beijing Zhongshan Golden Bridge Biotechnology Co., Ltd. Superoxide dismutase 1 (SOD1) and glutathione peroxidase 1 (GPX1) primers were synthesized by Shanghai Biological Engineering Company. 3-(4,5 dimethylthiazole-2yl)-2,5-diphenyl tetrazolium bromide thiazolyl blue (MTT) was purchased from Wuhan Ling fly Technology Co., Ltd. Dichlorofluorescin diacetate (DCFH-DA) was purchased from the Beyotime Institute of Biotechnology (Shanghai, China). All other reagents were purchased from Chinese suppliers.

### Cell culture

Highly differentiated PC12 cells, obtained from the Chinese Academy of Sciences Committee Type Culture Collection cell bank, were cultured in 100 mm plates in high-glucose Dulbecco’s modified Eagle’s medium (DMEM) supplemented with 10% (v/v) fetal bovine serum (FBS), 100 U/ml penicillin and 100 U/ml streptomycin. Cells were incubated at 37 °C in a humidified incubator (Model No. 3130; Forma Scientific) supplemented with 5% CO_2_ and cultured to 80–90% confluency before being passaging or use in experimentation.

### MTT assay to evaluate cell survival

Cells were plated at 1 × 10^4^ cells/well in 96 well plates, cultured, differentiated and treated as described above. Then, PC12 cells were treated with various concentrations of EGCG (2.5, 5, 10, 20 and 40 μmol/L) and/or MPP^+^ (125, 250, 500, 1000 and 2000 μmol/L) for 24 h. Then, treatment solutions were discarded, and cells were washed gently with PBS. MTT (20 μl) at 0.5 mg/ml and medium (200 μl) were then added to each well. After incubating for 4 h at 37 °C, with 5% CO_2_, the solution was removed, and 150 μl dimethyl sulfoxide was added to each well. The optical density (OD) was evaluated at 570 nm by a microplate reader after the precipitate in each well was dissolved for 10 min.

### Preparation of cell lysates

PC12 cells were washed several times with ice-cold phosphate buffered saline (PBS), resuspended in RIPA lysis buffer (50 mM Tris, pH 7.4, 150 mM NaCl, 1% Triton X-100, 1% sodium deoxycholate, 0.1% sodium dodecyl sulfate (SDS), 2 mM sodium orthovanadate, 25 mM sodium fluoride, 1 mM EDTA and 0.5 mM phenylmethanesulfonyl fluoride and incubated for 10 min on ice. Lysates were then centrifuged at 12,000 × *g* for 10 min at 4 °C.

### Detection of intracellular reactive oxygen species (ROS)

To measure ROS in differentiated PC12 cells produced by MPP^+^ treatment, we used a DCFH-DA assay. DCFH-DA is a fluorescent dye that crosses the cell membrane and is enzymatically hydrolyzed by intracellular esterases to non-fluorescent DCFH. Cells were plated at 4 × 10^5^ cells per 6 well dish. Differentiated PC12 cells were pretreated with 10 μmol/L EGCG for 1 h and then treated with 500 μM MPP^+^ for 24 h. Cells were then incubated with DCFH-DA at a final concentration of 10 μM in high-glucose DMEM without FBS for 20 min at 37 °C, washed three times with DMEM and analyzed using a FACSCalibur flow cytometer (Becton Dickinson, San Jose, CA, USA). For each analysis, 10,000 events were recorded.

### Western blot analysis

Thirty micrograms of protein were loaded per lane and resolved by 10% SDS-polyacrylamide gel electrophoresis for 90 min at 80 V. Separated proteins were transferred to polyvinylidene fluoride membranes (Millipore) for 2 h at 140 V with a Bradford reagent (Bio-Rad, CA). Membranes were blocked with 5% skim milk in PBS containing 0.05% Tween 20 for 1 h at room temperature. Then, membranes were incubated with primary antibodies against PGC-1α (Lot#: B2510, Santa Cruz), SIRT1 (Lot#: K0309, Santa Cruz) and β-actin. Proteins were detected with a horseradish peroxidase-coupled secondary antibody. Specific bands were visualized using an enhanced chemiluminescence detection kit (Cat#:: AR1170; Millipore,Billerica, Massachusetts,America).

### RNA isolation and real-time RT-PCR

Total RNA was isolated from PC12 cells using TRIzol reagent (Invitrogen, CA) according to the manufacturer’s protocol. Total RNA purity and integrity was confirmed by an ND-1000 NanoDrop (NanoDrop Technologies) and 2100 Bioanalyzer (Agilent). Real-time PCR was performed using an ABI prism 7500 HT sequence detection system (Applied Biosystems, Foster City, CA) based on the 59-nuclease assay [4] for the genes indicated and the housekeeping gene glyceraldehyde-3-phosphate dehydrogenase. Relative expression was calculated using the ΔΔCt method [5]. PCR amplification was carried out with cDNA equivalent to 10 ng starting mRNA using specific oligonucleotide primers for PGC-1α (forward, 5’-CAATGAATGCAGCGGTCTTA-3’, reverse, 5’-ACGTCTTTGTGGCTTTTGCT-3’), SOD1 (forward, 5’-CGGCTTCTGTCGTCTCCTTGCTT-3’, reverse, 5’-AACTGGTTCACCGCTTGCCTTCT-3’), GPX1 (forward, 5’-CGGACATCAGGAGAATGGCAAGA-3’, reverse 5’-AGGAAGGTAAAGAGCGGGTGAGC-3’) and β-actin (forward, 5’-GGAGATTACTGCCCTGGCTCCTA-3’, reverse 5’-GACTCATCGTACTCCTGCTTGCTG-3’).

### Statistical analysis

Data were analyzed and expressed as the mean ± standard error of the mean. Comparisons were made using analysis of variance and the Newman-Keuls multiple comparisons test. A value of P < 0.05 was considered statistically significant.

## Results

### EGCG suppresses decreased PC12 cell viability induced by MPP^+^

**Figure 1:**
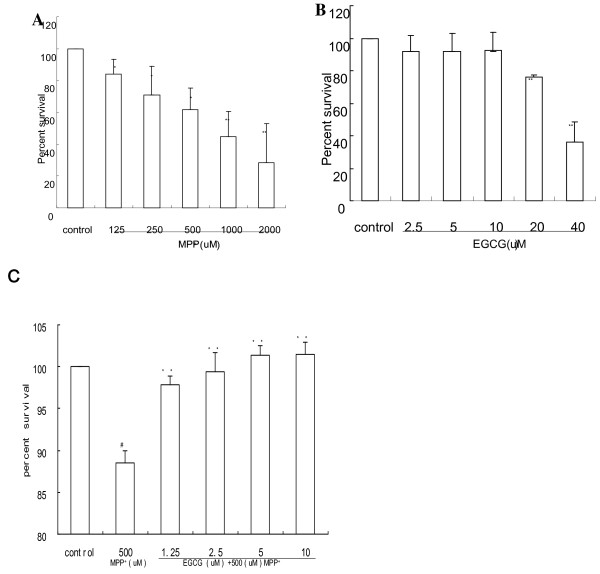
**EGCG and MPP**^**+**^**affect PC12 cell viability.(A)** MPP^+^-induced PC12 cell viability. MPP^+^ concentrations were 0, 125, 250, 500, 1000 and 2000 μmol/L (*P < 0.05;**P < 0.01 vs. vehicle control). **(B)** PC12 cell viability after EGCG treatment. EGCG concentrations were 0, 2.5, 5, 10, 20 and 40 μmol/L (**P < 0.01 vs. vehicle control). **(C)** Treatment with EGCG and then MPP^+^ affects PC12 cell viability. Groups were: control, 500 μmol/L MPP^+^, 500 μmol/L MPP^+^ + EGCG (1.25, 2.5, 5 or 10 μmol/L) (#P < 0.05 vs. vehicle control, **P < 0.01 vs. MPP^+^ treatment alone).

To evaluate the cell viability of differentiated PC12 cells after exposure to oxidative injury, PC12 cells were treated with various concentrations of MPP^+^ (125–2000 μmol/L) for 24 h. Different concentrations of MPP^+^ significantly decreased cell viability in a dose-dependent manner (Figure 1A). As an optimal MPP^+^ concentration, 500 μmol/L MPP^+^ was selected for subsequent experiments, because cell viability was deceased by 38.2% (P = 0.011) (Figure 1A). We investigated the neuroprotective effects of EGCG, which showed no cytotoxic effect at concentrations ranging from 2.5 to 10 μmol/L. However, at 20–40 μmol/L EGCG, PC12 cell viability was significantly attenuated (Figure 1B). PC12 cells were pretreated with 10 μmol/L EGCG for 1 h and then treated with 500 μmol/L MPP^+^ for 24 h. An MTT assay indicated that the cell viability of PC12 cells treated with EGCG and then MPP ^+^ was significantly increased by 12.92% (P < 0.05) compared with that of MPP^+^ treatment alone. However, there was no significant difference compared with the cell viability of the control (P > 0.05) (Figure 1C).

### EGCG attenuates intracellular ROS production by MPP^+^-induced oxidative stress

**Figure 2:**
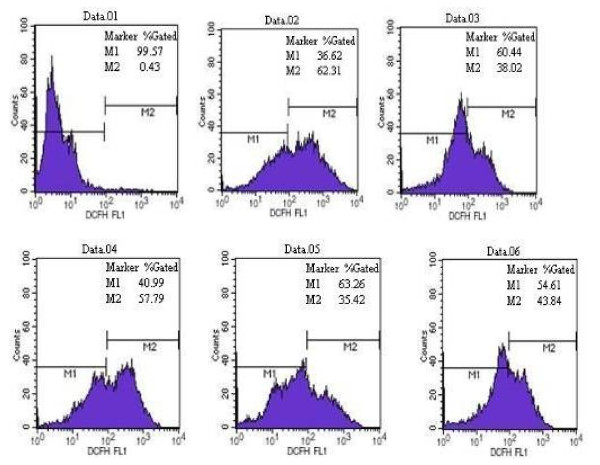
**EGCG attenuates intracellular ROS production after MPP**^**+**^**-induced oxidative stress.** PC12 cells were treated as indicated by the various groups. Data. 01: negative control, control group without DCFH-DA; Data.02: positive control, control group with DCFH-DA and rosup; Data.03: control group with DCFH-DA; Data.04: 500 μM MPP^+^; Data.05: 10 μM EGCG; Data.06: 500 μM MPP^+^ and EGCG 10 μM. Intracellular ROS production was determined by DCFH-DA fluorescence.

To measure intracellular ROS production in PC12 cells after MPP^+^ treatment and the effect of EGCG on ROS production, we used a DCFH-DA assay. As shown in Figure 2, MPP^+^ increased ROS production by 19.77%, compared with that in the control. Pretreatment with EGCG (10 μM) resulted in intracellular ROS production decreasing by 13.95%, compared with that of MPP^+^ treatment alone. EGCG alone also attenuated ROS production by 2.6%, compared with that in the control. These results indicated that EGCG treatment significantly suppressed intracellular ROS production in PC12 cells, compared with that in the control and MPP^+^ treatment alone. Therefore, EGCG showed a significant protective effect against MPP^+^-induced oxidative stress (Figure 2).

### PGC-1α and SIRT1 protein expression is enhanced by EGCG pretreatment of MPP^+^-treated PC12 cells

**Figure 3:**
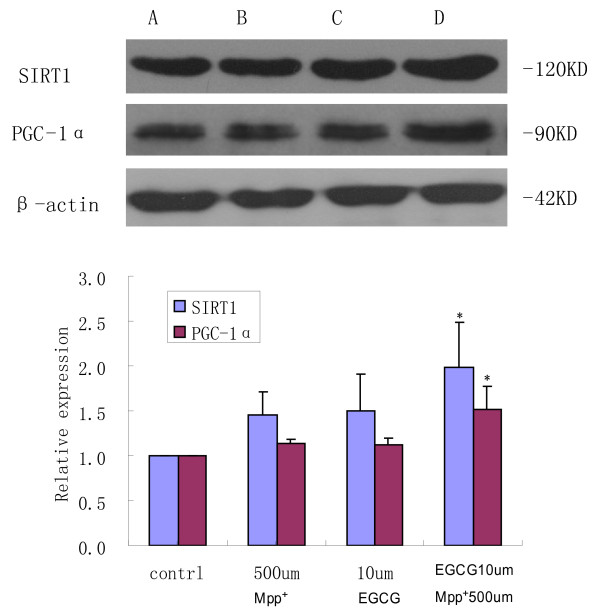
**Effect of MPP**^**+**^**and EGCG on PGC-1α and SIRT1 protein expression in PC12 cells.(A)** vehicle-only control, **(B)** MPP^+^ alone, MPP^+^ 500 μmol/L, **(C)** EGCG alone, 10 μmol/L EGCG, **(D)** 10 μmol/L EGCG + 500 μmol/L MPP^+^ (treatment with EGCG for 30 min, followed by MPP^+^ treatment). Results show that EGCG pretreatment could enhance PGC-1α and SIRT1 protein expression, and this up-regulation was higher than respective treatments with MPP^+^ and EGCG alone. β-actin was used as an internal reference. *P < 0.05 vs. vehicle-only control.

Western blot showed that there were protein bands at 120, 90 and 42 kDa (Figure 3). PGC-1α and SIRT1 protein levels were not obviously affected in PC12 cells treated with EGCG and then MPP^+^, compared with those in the control. PGC-1α protein expression was significantly elevated by 25.91% (P < 0.05), and SIRT1 protein expression was increased by 20.55% (P < 0.05) after treatment with EGCG and then MPP^+^, compared with that of MPP^+^ and EGCG treatments alone (Figure 3).

### PGC-1α, SOD1 and GPX1 mRNA levels are elevated in a time-dependent manner by EGCG pretreatment of MPP^+^-treated PC12 cells for 12 and 24 h

**Figure 4:**
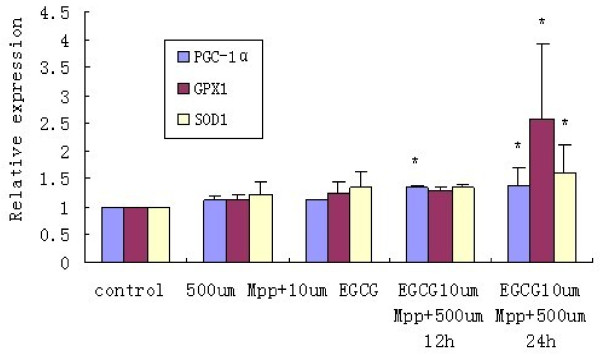
**Effect of MPP**^**+**^**and EGCG on PGC-1α, GPX1 and SOD1 mRNA expression in PC12 cells.** Groups were: control, 500 μmol/L MPP^+^, 10 μmol/L EGCG, 10 μmol/L EGCG +500 μmol/L MPP^+^ for 12 h (treatment with EGCG for 30 min, followed by MPP^+^ for 12 h), 10 μmol/L EGCG + 500 μmol/L MPP^+^ for 24 h (treatment with EGCG for 30 min, followed by MPP^+^ for 24 h). Results show that PGC-1α mRNA expression was obviously upregulated by 21.51% (P < 0.05), and SOD1 and GPX1 mRNA expression was slightly increased by treatment with EGCG and then MPP^+^ for 12 h. PGC-1αz SOD1 and GPX1 mRNA expression were increased by 25.17%, 40% and 146% (all P < 0.05), respectively, by treatment with EGCG and then MPP^+^ for 24 h,.*P < 0.05 vs. vehicle-only control.

Real-time PCR showed that PGC-1α, SOD1 and GPX1 mRNA expression was not significantly affected by MPP^+^ and EGCG treatments alone, compared with those in the control. PGC-1α mRNA expression was obviously upregulated by 21.51% (P < 0.05), SOD1 and GPX1 mRNA expression was slightly increased by 12.94% and 15.63% (P > 0.05), respectively, by treatment with EGCG and then MPP^+^ for 12 h. PGC-1α, SOD1 and GPX1 mRNA expression was increased by 25.17%, 40% and 146% (all P < 0.05), respectively, by treatment with EGCG and then MPP^+^ for 24 h, compared with those by MPP^+^ and EGCG treatments alone (Figure 4).

## Discussion

Drinking at least two cups of green tea a day can reduce the risk of Parkinson's disease [6]. Green tea extract contains polyphenols that have strong biological effects such as anti-oxidization, free radical scavenging, anti-apoptosis and inhibition of monoamine oxidase B. In addition, polyphenols can pass through the blood brain barrier and may be a drug candidate for treating neurodegenerative diseases [7]. Most of the benefits of green tea are due to the catechin EGCG, which is the most abundant and active catechin in green tea [8,9].

In drug-induced nerve degeneration research, MPP ^+^ has been widely used to study dopamine neurotoxicity, because it can lead to loss of dopaminergic neurons to induce Parkinson’s disease-like symptoms in animals [10]. MPP ^+^ is a mitochondrial complex I inhibitor and can cause an absence of ATP and loss of membrane potential in mitochondria, which leads to mitochondrial dysfunction, increased production of oxygen free radicals and cell damage [11]. PC12 cells derived from rat adrenal medulla pheochromocytoma, which are characterized by catecholamine synthesis, metabolism and transport system, are a cell model of Parkinson’s disease and have been widely used in neuroscience research [12].

A previous study found that EGCG pretreatment can significantly improve the survival of MPP^+^-treated PC12 cells in a dose-dependent manner. The protective effect of EGCG reached a plateau at 5 and 10 μmol/L, which is similar to the results of Mandel et al [13]. The dose-dependency of the protective effect of EGCG may be related to the cell type and high concentrations of MPP^+^-induced oxidative stress. Free radicals can generate unsaturated fatty acids, lipid peroxidation and oxidative damage of proteins and DNA, which leads to cell degeneration and death. ROS have multiple effects on cell function depending on the amount and subcellular location of ROS. Some studies have reported that ROS are involved in the apoptotic mechanism of MPP^+^-mediated neurotoxicity. As mentioned previously, our data also show that treatment with MPP^+^ results in a significant increase of ROS, and ROS activity assays indicate that pretreatment with EGCG significantly suppresses ROS generation in PC12 cells, compared with that in the control and MPP^+^-treated cells (Figure 2). We also found that EGCG shows a significant protective effect against MPP^+^-induced toxicity.

The expression of SOD1 and GPX1, a typical class of antioxidant enzymes, increases during oxidative stress, which can remove ROS and reduce damage by oxidative stress. PGC-1α, which is found in the anti-oxidative stress system, plays a key role in transcriptional regulation. PGC-1α is a multifunctional protein that can activate most nuclear receptors and also has a co-activation function for many transcription factors [14]. When neurons undergo oxidative stress, increased PGC-1α can regulate the cell response in which oxidant hydrogen peroxide increases the expression of PGC-1α, while the expression of antioxidant enzyme genes, such as SOD1, SOD2 and GPX1, significantly increase [15]. PGC-1α is activated by oxidative stress and induces the expression of antioxidant enzymes, which scavenge ROS and increase the antioxidant capacity. SIRT1 has been recently discovered as a class of nicotinamide adenine dinucleotide-dependent histone deacetylase and its activation can maintain deacetylation of PGC-1α to activate the combination of PGC-1α with chromatin to improve transcriptional activity. If PGC-lα is not bound to a transcription factor, the transcriptional activity is very low. However, transcriptional activity can be markedly elevated after SIRT1 and PGC-lα are bound to each other [16]. Attenuated intracellular SOD1 and GPX1 mRNA levels show that the protective effect of EGCG is related to scavenging intracellular oxygen free radicals. PGC-1α and SIRT1 protein levels are increased in PC12 cells, indicating that the antioxidant properties of EGCG are related to the expression levels of PGC-1α and SIRT1. PGC-1α, SOD1 and GPX1 mRNA levels are elevated in a time-dependent manner, indicating that the elevated SOD1 and GPX1 levels associated with PGC-1α may be a result of EGCG regulation of PGC-1α expression, which protects PC12 cells against MPP^+^-induced injury.

## Conclusions

EGCG, acting via the SIRT1/PGC-1α pathway, could increase the expression of antioxidant enzymes, remove free radicals and inhibit cell degeneration and death caused by MPP^+^. EGCG may activate other pathways to preserve PC12 cells against MPP^+^ damage, which requires further study. The antioxidant EGCG can play an effective protection role in the pathogenesis of Parkinson’s disease and is expected to become a therapeutic drug for treating Parkinson’s disease.

## Abbreviations

EGCG, (−)-Epigallocatechin-3-gallate; MMP+, -Methyl-4-phenyl-pyridine; PGC-1α, Peroxisomal proliferator-activated receptor- coactivator 1α; SIRT1, Silent mating type information regulation 2 homolog 1; SOD1, Uperoxide dismutase 1; GPX1, Glutathione peroxidase 1; ROS, Reactive oxygen species.

## Competing interests

The authors declare that they have no competing interests.

## Authors’ contributions

Qinyong Ye conceived and supervised the study, Linfeng Ye carried out immunoassays and real-time PCR, Xianjie Xu participated in cell culture, Bixia Huang participated in the DCFH-DA assay and helped to draft the manuscript, Xiaodong Zhang also helped to draft the manuscript, Yuangui Zhu and Xiaochun Chen also conceived the study. All authors read and approved the final manuscript.

## Pre-publication history

The pre-publication history for this paper can be accessed here:

http://www.biomedcentral.com/1472-6882/12/82/prepub
